# Rotator cuff repair with single row technique provides satisfying clinical results despite consistent MRI retear rate

**DOI:** 10.1186/s10195-022-00642-x

**Published:** 2022-05-04

**Authors:** Eugenio Vecchini, Matteo Ricci, Nicholas Elena, Luca Gasperotti, Andrea Cochetti, Bruno Magnan

**Affiliations:** 1 Department of Orthopedics and Trauma Surgery, University of Verona, Verona, Italy; 2 Department of Orthopedics, Solatrix Hospital, Rovereto, Italy

**Keywords:** Shoulder, Arthroscopy, Rotator cuff, MRI, Single-row repair

## Abstract

**Background:**

The number of shoulder arthroscopies is steadily increasing to treat glenohumeral joint disorders, among which the rotator cuff tear is the most common. The prevalence of this condition ranges from 13% to 37% in the general population without considering the number of asymptomatic patients. The gold standard procedure for rotator cuff repair is still undefined. The purpose of this study is to evaluate a population who underwent a single row (SR) rotator cuff repair and correlate their clinical results with MRI findings.

**Materials and methods:**

Sixty-seven consecutive rotator cuff procedures were retrospectively selected. All patients were diagnosed with a full-thickness rotator cuff tear and subsequently treated with an arthroscopic SR repair technique. Each patient was clinically assessed with the DASH questionnaire and the Constant–Murley Score to grade their satisfaction. Moreover, rotator cuff repair integrity was evaluated by MRI and graded using the Sugaya score.

**Results:**

Mean follow-up was 19.5 ± 5.7 months. The mean Constant score was 82.8 ± 13.0 points, with 55 patients reporting excellent results. No patient scored less than 30 points, which could be deemed as unsatisfying. Meanwhile, on the DASH questionnaire, 6.1% of our patients rated their clinical outcome as unsatisfying, whereas 75.8% rated their outcome as excellent. Postoperative MRI classified 45 patients (83.3%) as either Sugaya type I, II, or III, whereas 9 patients (16.7%) presented a Sugaya type IV consistent with a full-thickness cuff retear. Of these nine patients, five (55.6%) and three (33.3%) reported excellent results for the Constant score and DASH questionnaire, respectively. The Mann–Whitney test reported that the retear group had worse scores than the intact repaired cuff group for pain (8.3 ± 5.0 versus 13.1 ± 3.4), Constant Score (68.8 ± 18.5 versus 83.1 ± 11.6), and DASH (66.2 ± 22.1 versus 44.2 ± 14.9). Still, range of motion (ROM) differences were not significant, except for better forward flexion in the intact group (*p* < 0.039).

**Conclusions:**

Both groups with intact repaired and retorn cuffs showed improvement in their condition, but unexpectedly, there is no significant  correlation between patient satisfaction and rotator cuff integrity.

**Level of evidence:**

IV

## Introduction

Rotator cuff tear is the most common disorder in patients with shoulder problems. In the literature, the exact prevalence of this pathology is not well known since a large number of cases remain clinically asymptomatic. Many studies have tried to estimate the prevalence of rotator cuff disease in the adult population [1]. Recent studies report percentages from 13% to 37% of partial lesions in the general population and a prevalence of 30.3% of complete tears in the 60-year-old population [2]. In the past three decades, arthroscopic repair of the rotator cuff tear has become the gold standard intervention when symptoms do not improve with conservative treatment [3]. The two main surgical techniques performed to restore a cuff tear are the single row (SR) and the double row (DR) repair. Although several studies have demonstrated the biomechanical superiority of the DR compared with the SR method [4], there is no consensus regarding its preeminence in functional results. Still, the technique employed depends mostly on cuff tear size [5, 6].

Furthermore, rotator cuff integrity after the surgical procedure is assumed due to improved postoperative functional results being proven wrong for both techniques [7–10]. Nowadays, it is known that patients are likely to experience a retear, as demonstrated by long-term follow-up studies. However, there is still little consensus on the clinical results over time [10, 11].

The present study evaluates the clinical and radiological outcomes of 67 SR supraspinatus cuff repair procedures and investigates the correlation between postoperative clinical outcomes and MRI findings.

## Material and methods

### Patient selection

Patients who underwent arthroscopic rotator cuff repair between November 2016 and August 2018 were retrospectively selected. Inclusion criteria were: minimum 10 months follow-up and pre- or intraoperative diagnosis of a full-thickness supraspinatus tear atraumatic lesion. Patients who received other arthroscopic procedures, not including supraspinatus tendon repairs, such as isolated tenodesis or tenotomy of the long head of the biceps, labral repair, isolated subscapularis repair, subacromial decompression, and spacer, were excluded from the study. Other exclusion criteria were: recurrence in patients who had previously undergone rotator cuff repair surgery and traumatic lesion of the rotator cuff. In total, 131 patients were eligible for inclusion; however, we lost 31 subjects at follow-up, and 36 were either unable or unwilling to come to the institution for a clinical and MRI evaluation. Specifically, 23 patients felt satisfied with the result and did not find it necessary to have a clinical assessment; 5 moved out of town; and 8 suffered clinical conditions unrelated to the rotator cuff. Thus, 64 subjects (18 men and 46 women) and 67 rotator cuff repairs were finally included with a mean follow-up of 19.5 ± 5.7 months (range 12–28 months). The mean age was 62 years (range 33–73 years). Specifically, 57 out of 67 shoulders of the studied population had a full-thickness atraumatic lesion diagnosed before surgery. The remaining ten patients were diagnosed with a full-thickness tear during the surgical procedure. All patients gave informed consent, and the study was approved by the institutional review board (protocol number 65121).

### Surgical technique

All procedures were arthroscopically performed using the SR repair technique. All patients were placed under general anesthesia in lateral decubitus with standard posterior, lateral, and posterolateral arthroscopic portals. The glenohumeral joint was first evaluated, including the torn cuff, the articular capsule, the biceps tendon, and the subscapularis tendon. In 22 patients, the surgeon detected a damaged or hyperemic long head of the biceps tendon (LHB) with signs of inflammation, and in these cases, an LHB tenotomy was performed. The debridement of adherences and subacromial decompression with acromioplasty was standard procedure executed with a shaver or electrocautery in all the patients. The footprint of the entire supraspinatus tear was visualized, and the mobility of the rotator cuff was evaluated with a grasper. In all the procedures, a 5.0 mm Twin-Fix Ultra Suture Anchors (Smith and Nephew, Watford, UK) was used to repair the tendon on the footprint with a 45° angle between the anchor and the humeral tuberosity. The size of the tear was classified according to the Southern California Orthopedic Institute (SCOI) classification system: either small (C1 + C2) or large (C3 + C4), and the number of anchors implanted depended on the anterior–posterior extension of the torn cuff. Usually, a tear smaller than 3 cm required one anchor, while two or three anchors were implanted when it was larger than 3 cm [12].

Using a Scorpion Suture Passer (Arthrex, Naples, Florida, USA), the anchor’s threads were passed through the lateral margin of the ruptured tendon and sutured with a modified Mason–Allen suture.

Postoperative rehabilitation was carried out with the same protocol for all patients. Two days after surgery, they started passive flexion of the shoulder joint and pendulum exercises. For the first 4 weeks after surgery, an abduction sling was applied and removed only to perform active-assisted ROM exercises. From postoperative weeks 4 to 8, the brace was gradually removed, strengthening exercises of the rotator cuff were introduced, and ROM was increased. The physical therapist added scapular girdle muscle strengthening and capsular stretching after 6–8 weeks.

Patients were cleared for sports activity 6 months after surgery.

### Clinical evaluation

The Constant–Murley Score was measured for this retrospective study, and the DASH questionnaire was administered at final follow-up to evaluate clinical and functional outcomes.

The Constant–Murley Score is considered one of the most reliable scores to evaluate clinical and functional improvements after surgery [13]. It is a 100-point scoring system divided into four sections: two parts consider subjective aspects (pain and limitations of daily living activities); the other two parts of the score evaluate objective factors (range of motion and strength of the affected arm). The final score aggregates the four sections weighted as follows: 0–15 points for pain, 20 points for activities of daily living, 40 points for range of motion (ROM), and 25 points for strength of the affected upper limb. We used a visual analog scale (VAS) to estimate the patient’s pain, and the result was converted into a range from 0 to 15 to fit the Constant score. The examiners used a four-question questionnaire to evaluate the activity of daily living (ADL) limitations: during general daily activities, during free time, sleep disorders caused by the affected shoulder, and maximum range of motion without pain. Two independent examiners assessed each patient. Passive and active range of motion on the considered planes was singularly measured and then aggregated. Forward flexion was estimated by dividing the sagittal plane into six parts of 30° each, assigning 0–10 points according to the flexion achieved. Abduction was scored using the same method on the coronal plane, while internal and external rotation was calculated on the transversal plane. The final score was the mean of the results obtained by the two examiners. The Constant score classifies the outcome as unsatisfying for a total score of 30 points or less; discrete for scores between 30 and 39 points; good between 40 and 59; very good from 60 to 69; and excellent over 70 points.

The Disability of the Arm, Shoulder and Hand (DASH) score, introduced by the American Academy of Orthopedic Surgeons, is considered by many authors a reliable and validated questionnaire to report the after-surgery outcomes of rotator cuff repair [14]. This score is composed of 30 items evaluating the amount of disability reported by patients during some of their activities of daily living after arthroscopic rotator cuff repair. We awarded 1–5 points, depending on the increased disability experienced, ranging from a minimum of 27 to a maximum of 150. Al least 27 out of 30 questions must be completed to validate the questionnaire.

The DASH score parameters investigate physical symptoms (pain, decreased sensitivity, weakness, upper limb stiffness) and social–psychological status (limitations to activity of daily living and social discomfort related to the operated arm).

### MRI examinations

Imaging is essential to evaluate muscle–tendon integrity. In fact, clinical scores, like the Constant score, in most cases have shown no differences between patients with an intact rotator cuff and patients with a full-thickness tendon tear [15].

According to the literature, magnetic resonance is the gold standard for evaluating tendon integrity. The Sugaya score is the most applied and reliable classification to evaluate postoperative structural rotator cuff outcomes [16, 17]. Images were obtained using different MRI devices. Nevertheless, every MRI had T1-weighted images with sagittal, coronal, and axial views and T2-weighted images on oblique coronal, oblique sagittal, and axial sections.

In our study, 51 patients and 54 shoulders underwent MRI examination, whereas 13 patients refused MRI because of claustrophobia or concurrent health issues.

Two examiners separately evaluated the MRIs by measuring the acromial–humeral interval (AHI) and the extension of tendon retraction and signal intensity in T2-weighted images. For every MRI, the examiners gave a score from 1 to 5 points, according to the Sugaya classification. Both examiners were unaware of the radiologist’s report to avoid possible biases. As a result, 50 images had the same score for both examiners; on the other hand, four MRIs presented discordant results; in these cases, a third examiner’s opinion was requested.

**Fig. 1:**
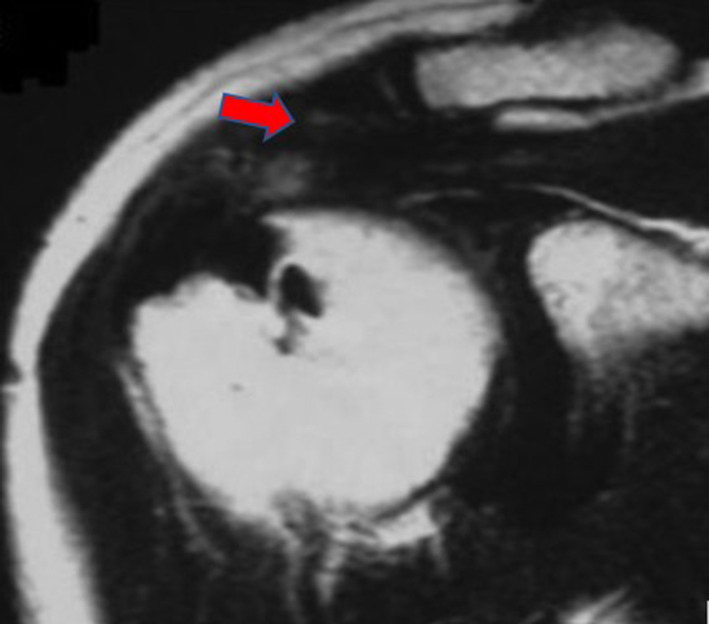
MRI of supraspinatus tear Sugaya type II

**Fig. 2:**
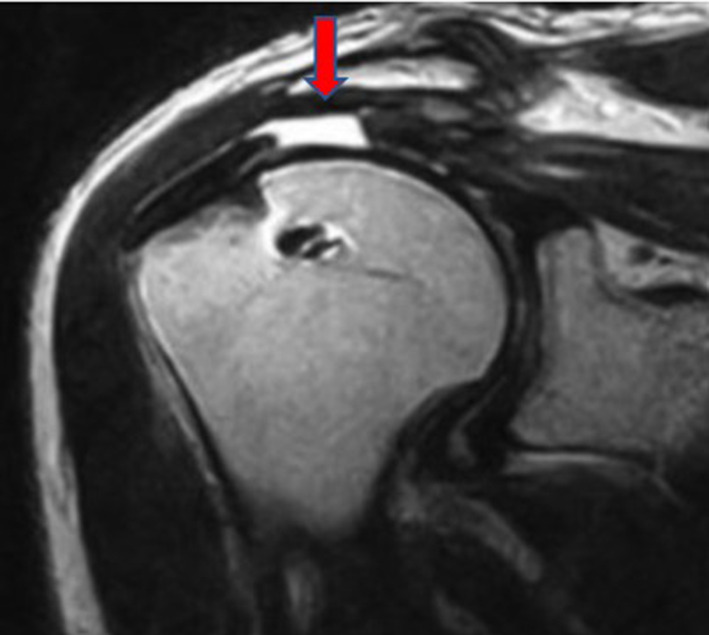
MRI of supraspinatus tear Sugaya type IV

**Fig. 3:**
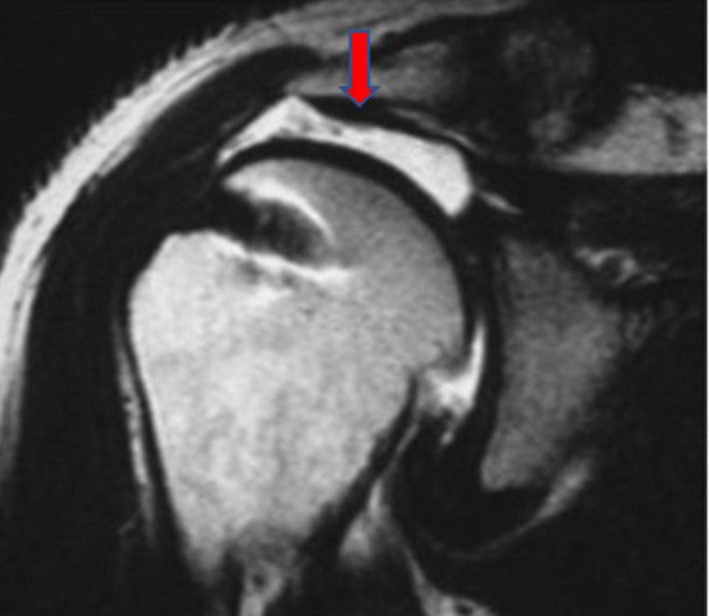
Example of MRI Sugaya type V of a non-enrolled patient

AHI is the minimum distance between the humeral head and the acromion on coronal and oblique sagittal T2-weighted images. Using oblique sagittal, oblique coronal, and transverse T2-weighted MRI images, cuff integrity was classified into one of the five categories of the Sugaya score: type I correlates to cuffs presenting sufficient thickness and a homogeneous tendon (low signal on T2 images); type II to images with sufficient thickness but partial high intensity from within the tendon (Fig. 1); type III to cuffs with insufficient thickness without discontinuity; type IV to minor discontinuity on more than one slice (Fig. 2), suggesting a small tear; type V to major discontinuity, suggesting a moderate or large tear (Fig. 3). In addition, the Sugaya classification was simplified, dividing the images into two groups: the first group included Sugaya type I, type II, and type III in which the cuffs were considered intact, differing only in thickness; a second group included Sugaya type IV and type V, in which rotator cuffs presented a full-thickness tear. According to this simplified classification, in our study, we diagnosed as retears of the rotator cuff only Sugaya types IV and V.

### Statistical analysis

Statistical analysis was performed using the Graph Pad Prism 8 software for Windows. Results are expressed as mean ± standard deviation, range, absolute frequency, and relative frequency. The normality and equal variance assumptions were assessed using the Shapiro–Wilk and Kolmogorov–Smirnov tests. The nonparametric Mann–Whitney test was used to compare final Constant score, pain, abduction, forward flexion, internal rotation, external rotation, strength, age, and final DASH score between patients with rotator cuff retear and patients with intact rotator cuff. *t*-Test was used to compare the Constant score and DASH scores between those who underwent biceps tenotomy and patients with intact CLB and between patients with a small tear (C1 or C2) and those with a large lesion (C3, C4). We adopted the Spearman *r* test to assess for a correlation between age and Sugaya level. Chi-squared test was used to compare independent variables between patients grouped by gender, smoking history, lesion laterality, and metabolic diseases, such as diabetes and hypercholesterolemia, and to compare retear rate between patients with a small tear (C1, C2) and patients with a large lesion (C3, C4).

## Results

### Clinical results

**Fig. 4:**
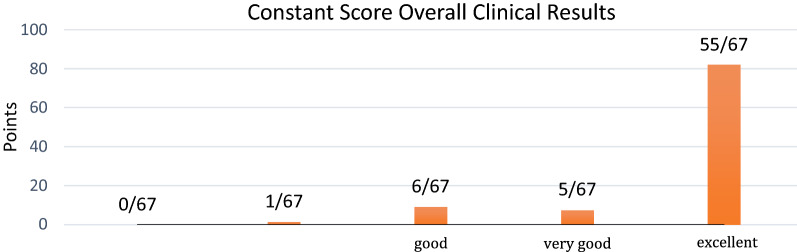
Constant score overall clinical results

Sixty-four patients and 67 shoulders were clinically evaluated. At final follow-up, the mean Constant score was 82.8 ± 13.0 points, with a minimum of 35 and a maximum of 98. No patients reported an unsatisfying final Constant score under 30 points. Only one patient (1.5%) reported a discrete final score, between 30 and 39 points. In six patients (9.0%), the score was classified as good, and five patients (7.5%) scored as very good, between 60 and 69 points. Finally, 55 patients (82.1%) obtained over 70 final points, reporting an excellent Constant score (Fig. 4 and Table 1). The mean postoperative pain was 12.4 ± 4.3 points; the mean ADL was 3.8 ± 3.0 points. Mean muscular strength was 21.5 ± 4.6 points. The ROM was divided into four categories showing a mean score for forward flexion of 9.5 ± 1.2 points (171.0–21.6°), external rotation 8.9 ± 2.2 (160.7 ± 38.9°), abduction 9.4 ± 1.5 (168.3° ± 27.5°), and internal rotation 7.9 ± 16 points (Table 1).

**Table 1:** Constant score results for each evaluated section

Parameter	Evaluation	Measure	Results [mean ± standard deviation (range)]
Pain	Mean	Point	12.4 ± 4.3 (0–15)
ADL	Work, free time, sleep	Point	3.8 ± 3.0 (0–8)
ROM	Forward flexion	Point Degree	9.5 ± 1.2 (6–10) 171 ± 21.6° (91–180°)
	Abduction	Point Degree	9.4 ± 1.5 (4–10) 168 ± 27.5° (61–180°)
	External rotation	Point Degree	8.9 ± 2.2 (0–10) 161 ± 38.9° (0–180°)
	Internal rotation	Point	7.9 ± 1.6 (4–10)
Strength	Mean (kg) × 2	Point	21.5 ± 4.6 (10–25)
Total			82.8 ± 13.0 (35–98)

The DASH score was recorded in 66 cases. The DASH questionnaire was not considered in one patient owing to a traumatic distal radioulnar joint injury at follow-up time, influencing more than three items of the score. The DASH score averaged 45.0 ± 17.3 points in the studied group, with a minimum final score of 30 and a maximum of 95. To express the results in percentage and facilitate comprehension, we converted the final score with the following formula: (sum of answers − 1/number of answers) × 25. The mean DASH score was 12.1 ± 14.0 points, with a minimum of 0 and a maximum of 54.2 points. Using the minimum clinically important difference (MCID) [18], patients’ results were divided into subcategories of 15 points each. Fifty questionnaires (75.8%) scored less than 15 points, considered an excellent score; seven patients (10.6%) scored between 15 and 30 final points, regarded as good, while five questionnaires (7.6%) reported scores between 30 and 45 points and four questionnaires (6.1%) scored more than 45 final points, considered as unsatisfying results (Table 2).

**Table 2:** Results of DASH score categorized by item with relative scores (mean ± standard deviation)

Dash score			
Item	Mean ± standard deviation (range)	Item	Mean ± standard deviation (range)
(1) Unscrew the lid of a jar	1.5 ± 1.2 (1–5)	(16) Use a knife to cut food	1.1 ± 0.8 (1–3)
(2) Write	1.2 ± 0.9 (1–3)	(17) Recreational activities with little effort	1.2 ± 0.9 (1–5)
(3) Turn a key	1.4 ± 0.9 (1–3)	(18) Recreational activities in which strength is used	1.4 ± 1.1 (1–5)
(4) Prepare a meal	1.4 ± 1.0 (1–5)	(19) Recreational activities with free arm movement	1.6 ± 1.1 (1–5)
(5) Open a heavy door by pushing	1.8 ± 1.2 (1–5)	(20) Capacity of moving	1.3 ± 1.0 (1–5)
(6) Place an object above your head	2.1 ± 1.6 (1–5)	(21) Sexual activity	–
(7) Do heavy housework (clean glass)	2.0 ± 1.2 (1–5)	(22) Everyday activities with friends	1.4 ± 1.0 (1–5)
(8) Do garden work	1.8 ± 1.2 (1–5)	(23) Work or daily activities	1.4 ± 1.1 (1–5)
(9) Make your bed	1.2 ± 1.1 (1–3)	(24) Shoulder pain	1.8 ± 0.9 (1–4)
(10) Carry the shopping bag	2.2 ± 1.1 (1–5)	(25) Pain during any activity	1.8 ± 0.9 (1–4)
(11) Carry heavy objects	2.3 ± 1.2 (1–5)	(26) Shoulder tingling	1.6 ± 0.8 (1–4)
(12) Change a light bulb	2.3 ± 1.5 (1–5)	(27) Shoulder weakness	1.4 ± 1.1 (1–5)
(13) Wash and dry your hair	2.1 ± 1.2 (1–5)	(28) Shoulder rigidity	1.3 ± 1.0 (1–3)
(14) Wash your back	2.1 ± 1.2 (1–5)	(29) Difficulty sleeping	1.5 ± 0.9 (1–5)
(15) Pull on a sweater	1.4 ± 0.9 (1–5)	(30) Feeling less confident or helpful	1.2 ± 1.1 (1–3)
Total (min 30–max 95)		45.0 ± 17.3 (30–95)	

We subdivided our patients into two groups: those who underwent a biceps tenotomy concurrently with a supraspinatus repair and those who did not. Afterward, we analyzed the data of the two groups separately. Patients with concurrent biceps tenotomy had a mean Constant score of 83.0 ± 11.5, a mean DASH score of 47.3 ± 19.3, and a VAS of 2.4. Whereas patients who presented with a healthy biceps tendon scored an average Constant of 82.7 ± 13.9, a mean DASH of 43.8 ± 16.2, and a VAS of 1.86. Comparing the results, we found a non-statistically significant difference between the two groups in term of Constant score (*p*-value 0.92) and DASH score (*p*-value 0.43).

Well aware of the size difference of the rotator cuff lesion, we also categorized the patients on the basis of their lesion size, i.e., 44 patients with an intraoperative small tear (C1 + C2) and 23 with a large rotator cuff lesion (C3 + C4), according to the SCOI classification [11], and analyzed the postoperative Constant and DASH scores of the two groups. The mean Constant score of patients with a small tear was 82.6 ± 14.3, and the mean DASH score was 45.9 ± 18.0. The mean Constant score of the group with a large tear was 83.2 ± 10.6, with a mean DASH score of 43.4 ± 16.1. Comparing functional results revealed no statistical differences in terms of Constant score (*p*-value 0.86) and DASH score (*p*-value 0.58) between the two groups. Furthermore, we separately analyzed the retear rate difference on MRI between patients with small tears and patients with large tears. Among patients with small tears, 13.8% had retear, while 22.2% of those with large cuff tears had retear. The difference between the two groups was not statistically significant, with a *p*-value of 0.6.

### MRI results

The authors analyzed 54 shoulders with postoperative MRI. Two patients (3.7%) presented a rotator cuff classified as Sugaya type I, 28 patients (51.9%) a type II, and 15 patients (27.7%) a type III.

Nine (16.7%) had a type IV Sugaya score, while none had type V. Consequently, 45 arthroscopic repairs (83.3%) reported no full-thickness lesions at follow-up. Meanwhile, nine MRI evaluations (16.7%) showed a full-thickness retear of the supraspinatus (Table 3).

**Table 3:** Sugaya score results expressed as numerical values and percentage

Sugaya grade	Number of shoulders	Percentage (%)
Sugaya type I	2	3.7
Sugaya type II	28	51.9
Sugaya type III	15	27.7
Sugaya type IV	9	16.7
Sugaya type V	0	0.0
Number of shoulders studied	54	

Data from patients who presented a Sugaya IV (no enrolled patient had Sugaya type V) were analyzed separately from the others (Table 4). Hence, the mean final Constant score of those nine patients was 68.8 ± 18.5 points, with a minimum of 35 and a maximum of 92 points. Five patients out of 9 (55.6%) scored more than 70 points, which was considered an excellent result, three (33.3%) scored between 40 and 59 points, while only one patient (11.1%) had an average final score of 35. The mean DASH score of these patients was 66.2 ± 22.1 points, with a minimum of 35 and a maximum of 91. Converting the final scores to hundredths, the mean result was 30.2 ± 18.4 points with a minimum of 4.2 and a maximum of 54.2. Following the hundredth distribution, three patients (33.3%) reported less than 15 points, considered excellent; three patients (33.3%) had an average final score between 30 and 44 points; while three patients (33.3%) reported more than 45 points, considered an unsatisfying result.

**Table 4:** Constant Score results expressed as numerical values and percentage

Constant score	Number of patients	Relative percentage (%)
< 30	0	0
30–39	1	11.1
40–59	3	33.3
60–69	0	0
70–100	5	55.6

### Clinical differences between patients with retears and those with an intact rotator cuff

The Mann–Whitney test compared the retear group with those who had intact rotator cuff data for non-normally distributed values (Table 5). Patients with MRI signs of retear were found to be associated with lower final Constant scores (*p*-value < 0.05), higher pain levels (*p*-value < 0.05), and a lower forward flexion ROM (*p*-value < 0.05). However, abduction, external rotation, internal rotation, and strength differed significantly between the two groups. Furthermore, using the same method for comparing the DASH questionnaire results, it appeared that patients with retears reported a significantly higher final score (*p*-value < 0.05) compared with the group of patients with an intact rotator cuff. Even though all the values mentioned earlier were significantly better in patients with intact repaired cuffs, 88.9% of patients with a cuff retear still achieved good-to-excellent results in terms of Constant score and 33.3% achieved excellent results on DASH evaluation. We also considered the retear rate between patients with small and large cuff lesions to determine the possibility of selection bias due to different tear extensions. Retears occurred in 13.8% of patients with small tears (C1 + C2), and in 22.2% of patients with large cuff tears (C3 + C4). This difference was not statistically significant, with a *p*-value of 0.6.

**Table 5:** Differences between patients with intact cuff and patients with retear in our study

At final follow-up:	Intact cuff, *n* = 45	Retear, *n* = 9	*p-Value*
(1) Final Constant score			
Mean	83.1 ± 11.6 (57–98)	68.8 ± 18.5 (35–92)	0.018
Median	87 (*n* = 45)	71 (*n* = 9)	
(2) Strength			
Mean	22.0 ± 4.1 (10–25)	18.4 ± 6.2 (10–25)	0.116
Median	25 (*n* = 45)	20 (*n* = 9)	
(3) Abduction			
Mean	9.4 ± 1.5 (4–10)	8.4 ± 2.2 (4–10)	0.062
Median	10 (*n* = 45)	10 (*n* = 9)	
(4) External rotation			
Mean	8.9 ± 2.0 (4–10)	6.9 ± 3.6 (4–10)	0.054
Median	10 (*n* = 45)	8 (*n* = 9)	
(5) Internal rotation			
Mean	7.9 ± 1.7 (4–10)	7.6 ± 1.7 (4–10)	0.595
Median	8 (*n* = 45)	8 (*n* = 9)	
(6) Forward flexion			
Mean	9.6 ± 1.1 (6–10)	8.7 ± 1.7 (6–10)	0.039
Median	10 (*n* = 45)	10 (*n* = 9)	
(7) Pain			
Mean	13.1 ± 3.4 (0–15)	8.3 ± 5.0 (0–15)	0.002
Median	15 (*n* = 45)	10 (*n* = 9)	
(8) Final DASH score			
Mean	44.2 ± 14.9 (30–89)	66.2 ± 22.1 (35–91)	0.003
Median	40 (*n* = 44)	73 (*n* = 9)	

Although the outcomes of these patients could be considered a failure from a purely surgical point of view as there was no tendon continuity, they are still successful according to clinical evaluation.

## Discussion

The pathophysiological mechanisms of the rotator cuff lesion can be divided substantially into two groups: the first consists of patients who have chronic, multifactorial, and age-related tendon degeneration; the second group is represented by acute traumatic injuries, linked to sports or work-related overuse of the joint. In our study, the authors enrolled only patients with a chronic cuff lesion to standardize the diagnostic and therapeutic process.

The treatment of chronic degenerative rotator cuff lesions first involves a conservative approach, usually lasting 6 months for older and 3 months for younger patients, based on physical rehabilitation therapy, oral anti-inflammatory drugs, and infiltrative treatment with corticosteroids or hyaluronic acid [19–22]. If this approach turns out to be unsuccessful, the alternative is a surgical procedure to reduce painful symptoms and restore function [23, 24].

Arthroscopic repair of the rotator cuff has become a routine procedure, following the evolution of surgery toward increasingly less invasive interventions. Compared with other techniques, it shows several advantages: fewer postoperative complications, better evaluation of the intra-articular space, faster recovery, less postoperative pain, and shorter hospital stay [25–31]. On the other hand, the most significant disadvantages of arthroscopy are a longer learning curve for surgeons and the high cost of anchors, especially when multiple fixations are required.

Clinical outcomes are comparable among the multitude of arthroscopic techniques [32]. In fact, most authors reported clinical results of arthroscopic rotator cuff repair with the single row technique as good or excellent [33–37], with a success rate greater than 80% in terms of pain control, joint function restoration, and patient satisfaction. In our study, the outcomes are overall positive and in accordance with the literature. Most patients returned to a good or excellent level of autonomy after surgery, with a mean total DASH score of 45.0 ± 17.3 points. Moreover, recovery of ROM, strength, and decreased postoperative pain, with a mean Constant score of 82.8 ± 13.0 points, can be considered successful. Among these, no patient reported an unsatisfactory clinical score; only one (1.5%) showed a final score considered as “discrete”; and six patients (9.0%) had a “good” Constant score, while all the others scored as “very good” or “excellent.”

Despite advanced anchoring and fixing techniques, arthroscopic repairs of rotator cuff tears still produce high retear rates, which remains the most common concern. Multifactorial causes should be addressed (including age, smoking, metabolic disorders, size of the lesion, tendon quality, fatty tendon degeneration, and surgical technique) and considered [38, 39]. Literature reports the prevalence of recurrent lesions between 10% and 94% in medium-to-long-term radiological evaluation [39] (Table 6).

**Table 6:** Retear rate in studies using arthroscopic repair technique in literature and our study

Author	Number of patients	Follow-up	Surgical technique	Imaging	Retear rate (%)
Spennacchio et al. (2015) [40]	35	15 months	Single row	RMN	11.4
Carbonel et al. (2012) [34]	80	24 months	Single row	RMN	7.1
Carbone et al. (2018) [33]	32	12 months	Single row	RMN	20.0
Fink Barnes et al. (2017) [41]	86	15.8 months	Single row	RMN and ultrasound	33.7
Yoshida et al. (2016) [15]	52	24 months	Single row	RMN	12.0
Sugaya et al. (2005) [42]	39	35 months	Single row	RMN	25.6
Boileau et al. (2005) [36]	65	29 months	Single row	RMN	29.0
Pennington et al. (2009) [9]	44	20 months	Single row	RMN	20.5
Koh et al. (2007) [43]	24	27.1 months	Single row	RMN	16.7
Anderson et al. (2006) [44]	52	30 months	Single row	RMN	16.7
Heubeber et al. (2007) [45]	30	18 months	Single row	RMN	42.0
**Vecchini et al. (2021)**	**54**	**19.5** months	**Single row**	**RMN**	**16.7**

Bold value indicate to make our study being easily recognible between others in the table

The percentage of rotator cuff retear determined in this study is in accordance with literature using the same surgical SR technique. Furthermore, we should mention that, within the retear group, no patient presented a Type V Sugaya score.

The two most common arthroscopic rotator cuff repair techniques are SR and DR. The DR technique has biomechanical advantages. For instance, the DR suture provides a significantly more pressurized contact zone and covers a larger area of the original footprint than the SR technique [46–49]. Nevertheless, even with the evidence of a potential anatomical and biomechanical improvement with the DR technique, there is still no clinical proof of the superiority over the other [7]. Three Level I studies by Franceschi et al. [50], Burks et al. [51], and Grasso et al. [52] demonstrated no significant difference in outcome scores, respectively UCLA, ASES, Constant and DASH are clinical scores each. Franceschi et al. [50] used UCLA score, Burk et al. [51] used UCLA, ASES, Constant Score and Grasso et al. [52] used DASH score to valuate patient's clinical outcome.

Russel et al. performed a meta-analysis to assess for correlation between structural integrity of the rotator cuff and patient outcome. Despite significant differences in strength, forward flexion, external rotation, and abduction on the scapular plane between retear and intact rotator cuff groups, the results did not show a significant correlation between structural integrity and overall Constant score [53]. The only significant correlation was between arm abduction, strength in the scapular plane, and Sugaya classification of rotator cuff integrity. This difference can be attributed to the supraspinatus muscle being the most involved in the abduction. Yoshida et al. show that the Sugaya classification is reliable and can predict the strength of abduction motion in the scapular plane [16].

Our study found a significant difference (*p*-value < 0.05) in terms of final Constant scores between patients with a recurrent lesion and patients with cuff integrity. Finally, applying the Mann–Whitney test to all the Constant score parameters revealed that patients with MRI evidence of cuff retear showed, on average, higher pain levels and a lower score in forward limb flexion. At the same time, there appeared to be no difference in external rotation, internal rotation, or strength during abduction.

A limitation of this study is the lack of preoperative data to precisely assess clinical improvement following repair. In addition, even though the number of the evaluated subjects is comparable to previously mentioned studies, a second limitation is the relatively small and heterogeneous pool of patients. Still, we did not observe any statistical significance by dividing our patients according to the size of lesion or to the treatment performed (biceps tenotomy or not).

Lastly, we did not keep track of supraspinatus retraction and fatty infiltration on preoperative MRI.

### Conclusion

Even though retears proved to be frequent, both groups with intact repaired and retorn cuff improved their condition, with most of the clinically assessed scores being significantly better in the intact cuff group. Thus, there is no significant correlation between unexpectedly patient satisfaction and rotator cuff integrity.

## Data Availability

The dataset generated is not publicly available owing to the risk of compromising patients’ privacy but is available from the corresponding author on reasonable request.

## Abbreviations

SR: Single row
DR: Double row
LHB: Long head of the biceps tendon
ROM: Range of motion
VAS: Visual analog scale
ADL: Activity of daily living
DASH score: Disability of the Arm, Shoulder and Hand
AHI: Acromial–humeral interval
MCID: Minimum clinically important difference
